# Endothelial Microparticles as Potential Biomarkers in the Assessment of Endothelial Dysfunction in Hypercholesterolemia

**DOI:** 10.3390/medicina58060824

**Published:** 2022-06-19

**Authors:** Nik Nor Izah Nik Ibrahim, Razlina Abdul Rahman, Maryam Azlan, Aniza Abd Aziz, Aida Hanum Ghulam Rasool

**Affiliations:** 1 Department of Pharmacology, School of Medical Sciences, Health Campus, Universiti Sains Malaysia, Kota Bharu 16150, Kelantan, Malaysia; aidakb@usm.my; 2 Hospital USM, Health Campus, Universiti Sains Malaysia, Kota Bharu 16150, Kelantan, Malaysia; razlina@usm.my; 3 Department of Family Medicine, School of Medical Sciences, Health Campus, Universiti Sains Malaysia, Kota Bharu 16150, Kelantan, Malaysia; 4 School of Health Sciences, Health Campus, Universiti Sains Malaysia, Kota Bharu 16150, Kelantan, Malaysia; maryamazlan@usm.my; 5 Faculty of Medicine, Universiti Sultan Zainal Abidin, Kuala Terengganu 20400, Terengganu, Malaysia; anizaabdaziz@unisza.edu.my

**Keywords:** microparticles, microvesicles, endothelial function, hypercholesterolemia, vasodilation

## Abstract

*Background and Objectives*: Endothelial microparticles (EMP) particularly CD31^+^/42^−^/AV^+^, CD144^+^/AV^+^ and CD62e^+^/AV^+^ have been reported as having increased in cardiovascular-related diseases, making them potential biomarkers for endothelial dysfunction. This study aimed to compare these EMPs in patients with hypercholesterolemia and healthy controls and to correlate their levels with endothelium-dependent vasodilation (EDV) assessed via pulse wave analysis (PWA); an established method of assessing endothelial function. *Materials and Methods*: EMPs from 88 subjects (44 hypercholesterolemia patients and 44 controls) were quantified from whole blood using flow cytometry analysis. Endothelial function was determined using PWA combined with pharmacological challenge. *Results*: CD31^+^/42^−^/AV^+^ (3.45 ± 4.74 count/µL vs. 1.33 ± 4.40 count/µL; *p* = 0.03), CD144^+^/AV^+^ (7.37 ± 12.66 count/µL vs. 1.42 ± 1.71 count/µL; *p* = 0.003) and CD62e^+^/AV^+^ (57.16 ± 56.22 count/µL vs. 20.78 ± 11.04 count/µL; *p* < 0.001) were significantly elevated in the hypercholesterolemic group compared with the controls, respectively. There was a significant inverse moderate correlation between all circulating EMPs and EDV: CD31^+^/42^−^/AV^+^ (r = −0.36, *p* = 0.001), CD144^+^/AV^+^ (r = −0.37, *p* = 0.001) and CD62e^+^/AV^+^ (r = −0.35, *p* = 0.002). *Conclusions*: All EMPs were raised in the patients with hypercholesterolemia, and these values correlated with the established method of assessing endothelial function.

## 1. Introduction

Atherosclerotic cardiovascular disease (ASCVD) remains the major cause of morbidity and mortality worldwide. It is a multifactorial disease involving several complex pathways implicating glucose and lipid metabolism as well as risk factors such as hypertension, diabetes mellitus, dyslipidemia, obesity and smoking. These risk factors are often associated with enhanced oxidative stress, a contributor to endothelial dysfunction [1,2,3,4,5]. Endothelial dysfunction is an independent cardiovascular risk predictor and has been shown to precede atherosclerosis [6,7] It results from an imbalance of vasodilators and vasoconstrictors, which disrupts the homeostasis of the vessel. Detecting it at an early stage before any clinical manifestations develop would be a cost-effective preventive strategy. A growing amount of evidence has recently indicated that endothelial microparticles (EMPs)—also termed endothelial microvesicles—may have a potential role as surrogate biomarkers for endothelial dysfunction [8,9,10,11,12,13,14]. Endothelial microparticles are vesicles measuring 0.1–1 µm that shed from the cell surface of the endothelium in response to cell activation or apoptosis. They carry endothelial proteins, such as cadherin and adhesion molecules, which could be identified based on the presence of certain cell surface clusters of differentiation (CD). These cell surface antigens are identified using specific antibodies and are commonly detected using flow cytometry. Some methods also include the detection of phosphatidylserine with phosphatidylserine ligands, such as annexin V. Phosphatidylserine is a phospholipid present on the inner surface of cells that will also be exposed during apoptosis or activation [15]. Clinically, raised EMP levels have been shown to be associated with cardiovascular and metabolic diseases, such as atherosclerosis [16], hypertension [11,17,18], acute coronary syndrome [14,19], stroke [20], diabetes [21,22], and obesity [23]. CD31, CD62e and CD144, with or without double labelling with annexin V combination, are among the most extensively studied cell surface antigens commonly associated with endothelial dysfunction. It has been suggested that measuring EMP levels as biomarkers could be an easy and informative way to assess endothelial function, which, in turn, may then help in early detection and patient stratification [8,12,24,25].

The currently available non-invasive methods of assessing endothelial function include forearm blood flow (FBF) via plethysmography or the less invasive flow mediated dilatation (FMD) and pulse wave analysis (PWA) [10,26]. These are functional assessments based on the endothelium-dependent vasodilating action of endothelium-derived nitric oxide (NO) in a healthy endothelium. Reduced endothelium-dependent vasodilation (EDV) but unchanged endothelium-independent vasodilation (EIV) indicates impaired endothelial function. However, EDV assessments have some drawbacks, as the methods used may either be operator-dependent, tedious, or require drug administrations and thus may not be suitable for implementation in the clinical setting [10,26]. EMP detection may provide a complementary or perhaps alternative and simpler method for assessing endothelial function. However, it needs to be determined whether EMP levels correlate with those found via other already established methods of assessment before their establishment as surrogate biomarkers. Moreover, there has yet to be consensus regarding the method of EMP detection, with several different cell surface antigens being investigated in different studies.

Several reports have shown some correlations between EMP levels and functional assessments of endothelial function including FBF via plethysmography and FMD [17,23,27,28]. To the best of our knowledge, there have been no studies to date that correlate the levels of EMP and EDV assessed by PWA. There have also been no reports assessing all three EMPs double labelled with annexin V, particularly in patients with hypercholesterolemia. Thus, the aims of this paper are: (1) to evaluate the circulating EMP levels—namely CD31^+^/42^−^ (PECAM-1), CD144 (VE Cadherin), and CD62e (E-selectin)—in combination with annexin V labelling in a hypercholesterolemia cohort representing patients with endothelial dysfunction; and (2) to assess the relationship between EMP and EDV as assessed via PWA, which is an established method of assessing endothelial function. In this study, patients with hypercholesterolemia were chosen as this group of subjects are well known to have endothelial dysfunction [29,30].

## 2. Materials and Methods

### 2.1. Design and Participants

This study was approved by the Human Research Ethics Committee of Universiti Sains Malaysia (JEPeM-USM) (Protocol code: USM/JEPeM/16030100), and all subjects provided written informed consent according to the Declaration of Helsinki. This was a cross sectional study comparing 44 newly diagnosed and untreated patients with hypercholesterolemia aged ≥35 years with low-density lipoprotein (LDL)-cholesterol ≥4.1 mmol/L against 44 normal controls. Pregnant subjects and subjects who were already undergoing treatment for hyperlipidemia, or any treatment with vasoactive medication such as angiotensin-converting enzyme (ACE) inhibitors or angiotensin receptor blockers (ARB) were excluded from the study to eliminate any possible confounding effects of endothelial function improvement [31].

Before acceptance into the study, all potential subjects underwent a screening procedure that involved detailed history-taking and a physical examination. Five ml of venous blood was withdrawn from the antecubital vein of each participant for determining the fasting lipid profile (FLP) and EMP quantification.

### 2.2. EMP Quantification

Circulating EMPs were isolated from venous citrated blood drawn earlier via an atraumatic method using a 21-gauge needle. The samples were processed within 1 h according to recommendations by the International Society of Thrombosis and Hemostasis [32,33]. The method of isolation has been described elsewhere [34,35,36,37]. Briefly, the samples were centrifuged at 2500× *g* for 15 minutes at room temperature. The supernatant (plasma containing microparticles) was collected in a plastic tube and centrifuged again at 2500× *g* for 15 min, at room temperature. The speed of centrifugation was based on the recommendation by the International Society of Thrombosis and Hemostasis [34]. Fifty µL of platelet-poor plasma (PPP) was aliquoted and resuspended in 400 µL Annexin V binding buffer (diluted 1:10 in distilled water). Earlier, the buffer was sterile-filtered with 0.2 µm filter (Whatman, Maidstone, UK). Two µL each of fluorescent antibodies phycoerythrin (PE)-, peridinin-chlorophyll-protein (PerCP)-, and allophycocyanin (APC)- conjugated monoclonal antibody against CD31 (BD Biosciences, San Jose, CA, USA), CD42 (Biolegend, San Diego, CA, USA) and CD144(BD Biosciences, San Jose, CA, USA), respectively, were added, followed by incubation with 5 µL fluorescein isothiocyanate (FITC)-conjugated annexin V (BD Biosciences, San Jose, CA, USA) according to the manufacturer’s instructions. In a separate tube, 50 µL PPP was aliquoted and incubated with 2 µL PE- conjugated monoclonal antibody against 62e (BD Biosciences, San Jose, CA, USA), which was followed by incubation with 5 µL FITC-conjugated annexin V as above. This separate tube had to be prepared for monoclonal antibody against CD62e, as it was also PE-conjugated. Isotype matched (IgG) non-specific antibodies were added in a separate tube with the samples as negative controls. Stained samples were acquired using a BD FACSCantoII flow cytometer and BD FACSDIVA software (BD Biosciences, Erembodegem, Belgium). Nano polystyrene size standard beads 0.1–1.9 µm (Spherotech, Inc., Lake Forest, IL, USA) were used for size calibration. TruCount tubes (BD Biosciences, San Jose, CA, USA) with a known number of fluorescent beads were used for quantification of the microparticles. All EMPs were defined as particles less than 1.0 µm, stained positive for annexin V, and expressing surface antigens [35,38]; CD31^+^/42^−^ microparticles were defined as vesicles double positively labeled for CD31 and annexin V, but negatively labeled for CD42 (CD31^+^/42^−^/AV^+^), CD144 microparticles were defined as vesicles double positively labeled for CD144 and annexin V (CD144^+^/AV^+^) and CD62e microparticles were defined as vesicles double positively labeled for CD62e and annexin V (CD62e^+^/AV^+^). The data was analyzed using FCS Express 5 software (De Novo, Glendale, CA, USA).

The absolute count of cells was then calculated using the formula

$$\frac{\#\;of\;events\;in\;region\;containing\;cells}{\#\;of\;events\;in\;absolute\;count\;region}\times \frac{\#\;of\;beads\;per\;test}{volume}$$

### 2.3. Endothelial Function Assessment by PWA

Endothelial function assessment was performed at the Pharmacology Vascular Laboratory, School of Medical Sciences, Universiti Sains Malaysia after an overnight fast. The measurement of endothelial function was performed with the subjects lying supine; it was measured by the changes in the augmentation index (AIx) using SphygmoCor, (PWV Limited, Sydney, Australia) before and after pharmacological challenges. The method of assessment has been described elsewhere [39,40]. Briefly, after recording the baseline parameters of heart rate and blood pressure (Omron, Osaka, Japan), baseline AIx was recorded by placing the tonometer (Millar Instruments, Houston, TX, USA) over the radial pulsations. This was followed by the administration of a 500 µg sublingual nitroglycerine (Alpharma, Barnstable, UK) tablet for 3 min. Endothelium-independent vasodilation was defined as the maximum change in AIx after nitroglycerine administration. After a 30-min washout period [41,42], 2 × 200 µg of Ventolin Evohaler (GlaxoSmithKline, Marly-le-Roi, France) was administered via a spacer device (Trudel Medical Int., London, ON, Canada). Endothelium-dependent vasodilation was defined as the maximum change in AIx after salbutamol.

### 2.4. Data Analysis

Baseline characteristics, CD31^+^/42^−^/AV^+^, CD144^+^/AV^+^ and CD62e^+^/AV^+^ EMP levels and EDV between the patients with hypercholesterolemia and normal controls were compared using the independent *t*-test for continuous variables and Pearson’s chi-square test for categorical variables; an alpha (α) value of 0.05 was considered statistically significant. Data was presented as mean ± SD. An analysis of covariance (ANCOVA) was further performed and controlled for systolic blood pressure and body mass index ratio. The EMP values were log-transformed to achieve a normal distribution before the correlation analysis. Univariate correlation was performed with the Pearson correlation coefficient to evaluate the correlation between CD31^+^/42^−^/AV^+^, CD144^+^/AV^+^, and CD62e^+^/AV^+^ EMPs and EDV, EIV, and LDL-cholesterol levels. All analyses were performed using IBM SPSS (Statistical Packages for Social Science) 26.0 for windows (Inc., Chicago, IL, USA).

## 3. Results

The baseline characteristics of the study participants are shown in Table 1. By design, compared with the normal controls, the patients with hypercholesterolemia showed significantly higher levels of LDL-cholesterol. Triglyceride and total cholesterol were also significantly higher in the hypercholesterolemic group. Other anthropometric parameters did not significantly differ between the groups. As expected, EDV (2.26 ± 3.48%, vs., 7.47 ± 4.21%; *p* < 0.001) but not EIV (13.43 ± 3.75% vs. 14.59 ± 4.61%; *p* = 0.22) was significantly reduced in the hypercholesterolemic group when compared with the controls, respectively. The circulating EMPs, namely CD31^+^/42^−^/AV^+^ (3.45 ± 4.74 count/µL, vs., 1.33 ± 4.40 count/µL; *p* = 0.03), CD144^+^/AV^+^ (7.37 ± 12.66 count/µL, vs., 1.42 ± 1.71 count/µL; *p* = 0.003) and CD62e^+^/AV^+^ (57.16 ± 56.22 count/µL, vs., 20.78 ± 11.04 count/µL; *p* < 0.001) were significantly elevated in the hypercholesterolemic group compared with the controls. The differences persisted after controlling for possible confounders as shown in Table 2, for CD31^+^/42^−^/AV^+^, CD144^+^/AV^+^, and CD62e^+^/AV^+^, respectively.

**Figure 1:**
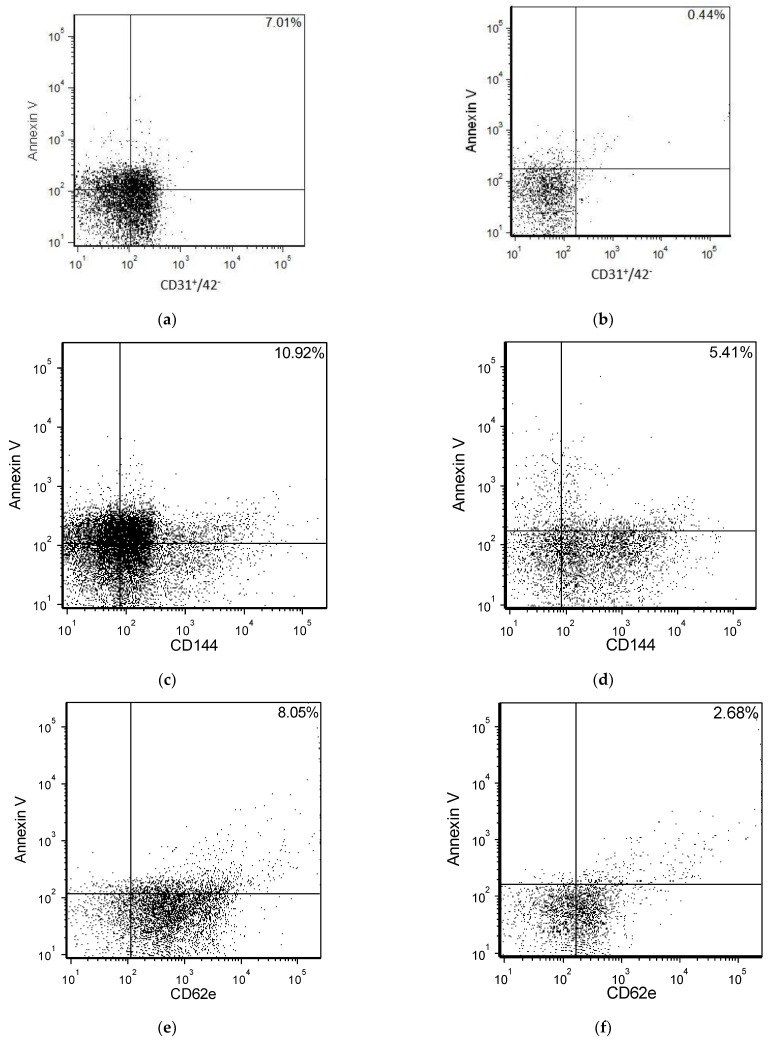
Representative FACS plots of EMP in plasma in one patient with hypercholesterolemia (**a**,**c**,**e**) and from one normal control (**b**,**d**,**f**). An increased CD31^+^/42^−^/AV^+^ (**a**) vs. (**b**), CD144^+^/AV^+^ (**c**) vs. (**d**) and CD62e^+^/AV^+^ (**e**) vs. (**f**) in plasma of patient with hypercholesterolemia in contrast to normal control.

Representative plots from the flow cytometry analysis that depict differences in the double-labelled EMPs with annexin V in the patients with hypercholesterolemia compared with the normal controls are shown in Figure 1.

**Figure 2:**
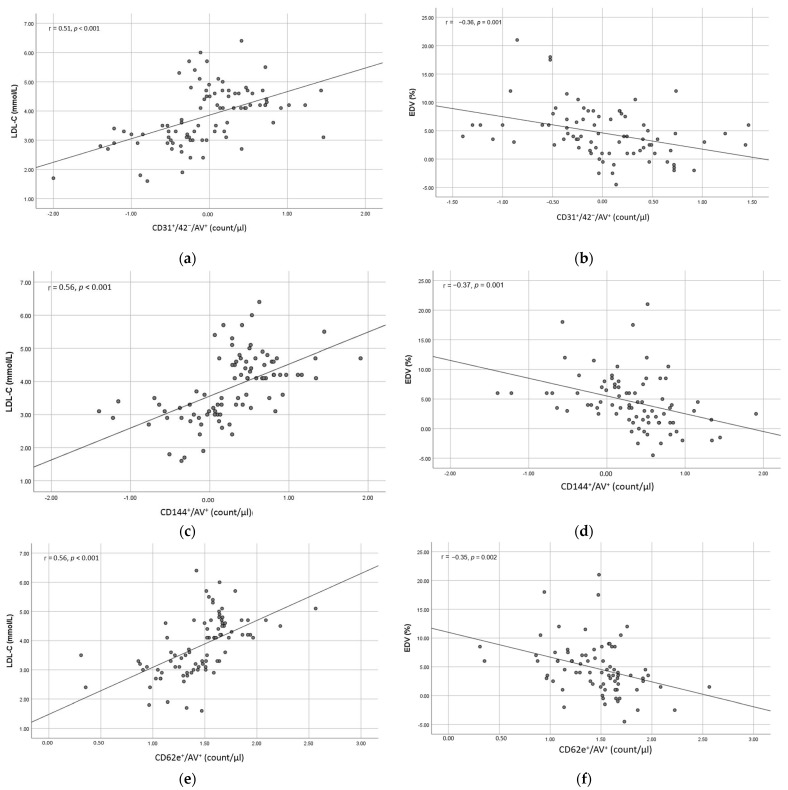
Correlation between LDL-cholesterol (LDL-C) (**left**), endothelium-dependent vasodilation (EDV) (**right**) and the absolute count of CD31^+^/42^−^/AV^+^ ((**a**) and (**b**) respectively), CD144^+^/AV^+^ ((**c**) and (**d**) respectively) and CD62 e^+^/AV^+^ ((**e**) and (**f**) respectively). The number of EMPs are log transformed (log base 10).

**Figure 3:**
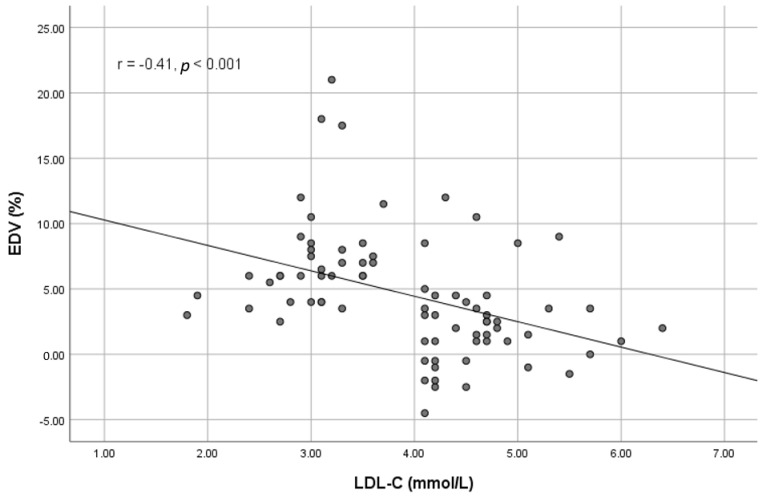
Correlation between endothelium-dependent vasodilation (EDV) and LDL-cholesterol.

A significant good direct correlation was observed between all circulating EMPs investigated and LDL-cholesterol; CD31^+^/42^−^/AV^+^ (r = 0.51, *p* < 0.001), CD144^+^/AV^+^ (r = 0.56, *p* < 0.001), and CD62e^+^/AV^+^ (r = 0.56, *p* < 0.001). There was also a significant inverse moderate correlation between all circulating EMPs investigated and EDV; CD31^+^/42^−^/AV^+^ (r = −0.36, *p* = 0.001), CD144^+^/AV^+^ (r = −0.37, *p* = 0.001), and CD62e^+^/AV^+^ (r = −0.35, *p* = 0.002) (Figure 2). No correlation was observed between the circulating EMPs and EIV: r = −0.037 (*p* = 0.74), r = −0.12 (*p* = 0.28) and r = −0.16 (*p* = 0.16) for CD31^+^/42^−^/AV^+^, CD144^+^/AV^+^, and CD62e^+^/AV^+^, respectively. A significant inverse correlation was also observed between EDV and LDL-cholesterol levels: r = −0.41, *p* < 0.001 (Figure 3).

## 4. Discussion

The key findings of this study were that: (a) the absolute counts of CD31^+^/42^−^, CD144, and CD62e, double-labelled with annexin V in patients with hypercholesterolemia were significantly raised when compared with age-matched normal controls; and (b) the EMP levels were inversely correlated with EDV, as assessed via PWA. To our knowledge, this is the first report on simultaneous assessments of CD31^+^/42^−^, CD144 and CD62e double-labelled with annexin V in hypercholesterolemic patients and also the first report on the correlations of the above EMP subtypes with the method of assessing endothelial function using PWA.

Hypercholesterolemia is known to be associated with endothelial dysfunction [29,30]; hence, the hypercholesterolemia cohort in this study represented patients with endothelial dysfunction. LDL-cholesterol alters the activity of endothelial nitric oxide synthase (eNOS). This results in a reduced NO bioavailability and an impaired vasodilatory function in the peripheral and coronary circulation [30,43]. Some novel mechanisms of endothelial dysfunction have also been reported. Carbonic anhydrase inhibitor (CA-I) has been shown to hamper endothelial cell permeability and results in endothelial cell apoptosis in vitro [44]. Another study showed that the administration of a high-fat meal resulted in an increase in tumor necrosis factor alpha (TNF-α), a cytokine that is associated with endothelial dysfunction [45].

Pulse wave analysis is one of the non-invasive techniques used to assess endothelial function. A previous study in hypercholesterolemia patients showed that the method correlated with FBF via plethysmography, an invasive and established method to assess endothelial function [39]. Our findings also showed the patients with hypercholesterolemia exhibited impaired endothelial function, as reflected by a reduction in EDV following the administration of inhaled salbutamol, but not in EIV following the administration of exogenous NO provided by sublingual nitroglycerine. These results were in agreement with previous findings, which showed the response to salbutamol, but not nitroglycerine, to be related to serum cholesterol [39]. However, this method is tedious and requires the administrations of two drugs, which may not be suitable for the clinical setting.

Our study showed elevated levels of CD31^+^/42^−^/AV^+^, CD144^+^/AV^+^, and CD62e^+^/AV^+^ in the hypercholesterolemic patients compared with the normal controls. Different combination sets of EMPs have been reported in different studies that support its role as a potential biomarker of endothelial dysfunction. Endothelial microparticles are defined according to their size and cell surface antigens detected. Several studies also include the detection of phosphatidylserine to ensure a more specific labelling [12,15,46]. The selection of the most specific markers to identify circulating EMP has been long debated. With the exception of CD144 and CD62e, which are endothelial specific, other markers, such as CD31 and CD105, are not exclusively endothelial. For instance, CD31 is also expressed by platelets, thus, the combination of multicolor antibodies (i.e., CD31^+^/42^−^ or CD31^+^/41^−^) has been suggested to improve its specificity [47,48,49].

In our study, we defined EMP as a double positive of a surface antigen (CD31^+^/42^−^, CD144 and CD62e, respectively) in combination with annexin V. Annexin V conjugates have been used in detecting EMPs, as they bind phosphatidylserine, which is a negatively charged phospholipid that is exposed on the cell surface of EMPs during vesiculation [15,50]. Microparticles and apoptotic bodies have been reported to externalize phosphatidylserine during activation or apoptosis, whereas another form of extracellular vesicle (i.e., exosomes) do not, under normal circumstances [48,51]. Size calibration with polystyrene microsphere beads and double-labelling with annexin V conjugates, which bind phosphatidylserine, may help distinguish microparticles and exosomes in our study. It has been suggested that annexin V staining is necessary to distinguish between true events and debris or precipitate, which may have the same size range as EMP [35]. However, the phosphatidylserine exposure process may not always occur in all microparticles. In the previous studies, it was reported to only have presented on approximately 60–86% of the cell surface of microparticles [46,52]. As a result, a significant amount of EMPs may not be detected when double-labelled with annexin V. As shown in our study, the absolute counts of CD31^+^/42^−^/AV^+^, CD144^+^/AV^+^ and CD62e^+^/AV^+^ in both groups were lower when compared to other studies without double-labelling with annexin V. However, the counts remained significantly different between the hypercholesterolemic and normal controls. It has been reported that, in diseased populations, such as cardiovascular disease, EMP levels may increase up to 10-fold due to cellular stress [12,53]. Our findings revealed that absolute counts ranged from less than a two-fold increase for CD62e^+^/AV^+^ and, a three-fold increase for CD31^+^/42^−^/AV^+^ to a seven-fold increase for CD144^+^/AV^+^ in the hypercholesterolemic groups compared with the controls. This may possibly be explained by the fact that our cohort consisted of only newly diagnosed hypercholesterolemic patients without complications from other cardiovascular diseases, and without other comorbidities—except for two hypertensives in the hypercholesterolemic group, and one in the control group.

The elevation of all circulating EMPs under investigation, namely CD31^+^/42^−^/AV^+^, CD144^+^/AV^+^, and CD62e^+^/AV^+^, in the hypercholesterolemic group supports their potential role as surrogate biomarkers of endothelial dysfunction. It was previously reported that the ratio of CD31^+^/42^−^ to endothelial progenitor cells, an index of endothelial injury, was raised in hypercholesterolemia [54]. Treatment with statin on the other hand, reduced microparticles shedding from the endothelium, platelets and inflammatory cells [30,46]. EMPs have been shown to increase with an increase in adiposity in both adults [23] and children [55]. Cholesterol and EMP levels have been shown to be very much linked, even though the actual mechanism is still unclear. Cholesterol is essential in the synthesis, release, and uptake of microparticles, including EMP [56]. In vitro, oxidized LDL has been shown to induce EMP release, which may possibly play a role in the atherosclerosis progression by causing monocyte recruitment and adhesion [57,58]. It is known that LDL-cholesterol and inflammatory reactions brought about by immune cells have been implicated in the pathogenesis of atherosclerosis. EMP release has also been associated with systemic inflammation and endothelial injury, which also takes place in the development of atherosclerosis. The release of EMPs increases cytokine production, and, vice versa, cytokine production increases the level of EMP [59]. An in vitro model of endothelial damage showed that EMP interferes with the endothelial repair process and angiogenesis, supporting the potential role of EMP as a biomarker of endothelial damage [60]. Besides being a biomarker, elevated levels of EMP have also been suggested to aggravate endothelial dysfunction, as this has been shown to reduce NO and increase superoxide levels in vitro [17,53]. Our study showed that EMPs and LDL-cholesterol were significantly and directly correlated. We have also shown that EDV determined via PWA was significantly and inversely correlated with LDL-cholesterol. Interestingly, the degree of correlation was better between all EMPs studied and LDL-cholesterol when compared with EDV and LDL-cholesterol. This finding may suggest a more promising role of EMP as surrogate biomarkers in endothelial dysfunction, particularly in hypercholesterolemia.

Finally, our study has shown that CD31^+^/42^−^/AV^+^, CD144^+^/AV^+^, and CD62e^+^/AV^+^ correlate with endothelial function, as reflected by EDV assessed via PWA. Previously, an inverse correlation between CD31^+^/42^−^ EMP levels and FBF via plethysmography has been established [17]. Another method of assessing microvascular endothelial function (i.e., laser Doppler flowmetry coupled with iontophoresis) has also been shown to correlate with EMP [33]. Several studies assessing the correlation between EMP levels and FMD have also clearly shown a similar inverse correlation. It was previously reported that there was an inverse correlation between CD31^+^/42^−^ and CD144 with FMD in patients with end-stage renal failure but no similar correlation for platelet or leukocyte microparticles [27]. Similar to our finding that no correlation was shown between EMP levels and EIV with exogenous NO provided by nitroglycerine, the authors also reported the endothelium-independent responses to exogenous NO were also not affected by EMP levels [27]. An inverse correlation between CD31^+^/42^−^ and FMD was also reported in an obese female population [23]. Another recent study also reported a similar inverse correlation between CD31^+^/42^−^, CD144 and CD62e with FMD in a hypertensive population [28]. Our study showed for the first time that the same subsets of EMPs were also correlated with another method of endothelial function assessment, i.e., using PWA. This and other inverse correlations between EMP values and the methods of endothelial function assessment thus support the potential role of EMPs as biomarkers of endothelial dysfunction.

We acknowledge some limitations in our study. First, some biochemical parameters such as fasting blood glucose and insulin blood levels were not determined in the subjects due to financial constraints, as we had to screen a large number of subjects to obtain sufficient numbers of newly diagnosed, untreated hypercholesterolemic subjects. However, a thorough history-taking was conducted prior to recruiting the study participants and none of our subjects were diabetics. Second, due to difficulties in recruiting adequate newly diagnosed hypercholesterolemic participants using overly stringent criteria, we had to also include a few smokers. There were only five smokers among the participants, and the numbers were not significantly different between the groups. Third, by using ultracentrifugation for the isolation of EMP, we could not exclude the possibility of contamination with cellular debris. However, it is unlikely that the level of contamination will affect the conclusion of this study, as appropriate surface markers and gating strategies have already been applied [61,62].

## 5. Conclusions

In summary, CD31^+^/42^−^/AV^+^, CD144^+^/AV^+^, and CD62e^+^/AV^+^ levels were raised in patients with hypercholesterolemia. These EMP levels were also inversely correlated with endothelial function as assessed via PWA, which is an established method to assess endothelial function. Thus, EMPs may have the potential to serve as biomarkers for endothelial dysfunction, particularly in patients with hypercholesterolemia.

## Figures and Tables

**Table 1:** Subjects characteristics.

Variable	Hypercholesterolaemia (*n* = 44)	Control (*n* = 44)	*p* Value
	Mean (SD)	Mean (SD)	
Age, y	43.48 (7.76)	42.30 (6.63)	0.45 ^a^
Male/female, *n*(%)/*n*(%)	15 (51.7)/29 (49.2)	14 (48.3)/30 (50.8)	0.82 ^b^
Smoker, *n*(%)	4 (9.1%)	1 (2.3%)	0.36 ^c^
Hypertension, *n*(%)	2 (4.5%)	1 (2.3%)	0.50 ^c^
BMI, kg/m^2^	27.63 (4.16)	25.99 (4.96)	0.10 ^a^
LDL, mmol/L	4.66 (0.56)	2.99 (0.51)	<0.001 ^a^
HDL, mmol/L	1.42 (0.29)	1.40 (0.33)	0.73 ^a^
Triglyceride, mmol/L	1.39 (0.50)	1.15 (0.59)	0.04 ^a^
Total Cholesterol, mmol/L	6.64 (0.74)	4.83 (0.82)	<0.001 ^a^
SBP, mmHg	122.90 (13.18)	119.26 (13.18)	0.18 ^a^
DBP, mmHg	76.57 (7.94)	74.91 (10.41)	0.40 ^a^
EIDV, %	13.43 (3.75)	14.59 (4.61)	0.22 ^a^
EDV, %	2.26 (3.48)	7.47 (4.21)	<0.001 ^a^
CD31^+^/42^−^/AV^+^, count/µL	3.45 (4.74)	1.33 (4.40)	0.03 ^a^
CD144^+^/AV^+^, count/µL	7.37 (12.66)	1.42 (1.71)	0.003 ^a^
CD62e^+^/AV^+^, count/µL	57.16 (56.22)	20.78 (11.04)	<0.001 ^a^

BMI = body mass index; LDL = low density lipoprotein; SBP = systolic blood pressure; DBP = diastolic blood pressure; EDV = endothelium-dependent vasodilation; EIV = endothelium-independent vasodilation. ^a^ Independent *t*-test; ^b^ Pearson’s Chi Square; ^c^ Fisher’s exact test.

**Table 2:** Comparison of endothelium microparticles (EMP) between hypercholesterolemia patients and healthy controls with and without adjustment of possible confounders.

Group	Mean (SD)		Mean Difference (95% CI)	*p* Value
	Hypercholesterolaemia (*n* = 44)	Control (*n* = 44)		
CD31^+^/42^−^/AV^+^	3.45 (4.74) ^a^	1.33 (4.40) ^a^	−2.12 (−4.06, −0.18)	0.032 ^b^
CD31^+^/42^−^/AV^+^ (adjusted)	3.41 (2.01, 4.81)	1.38 (−0.02, 2.78)	2.03 (0.03, 4.03) ^c^	0.047 ^d^
CD144^+^/AV^+^	7.37 (12.66) ^a^	1.42 (1.71) ^a^	−5.96 (−9.79, −2.13)	0.003
CD144^+^/AV^+^ (adjusted)	7.38 (4.62, 10.14)	1.41 (−1.35, 4.17)	5.96 (2.02, 9.91) ^c^	0.003 ^d^
CD62e^+^/AV^+^	57.16 (56.22) ^a^	20.78 (11.04) ^a^	36.38 (−53.56, −19.20)	<0.001
CD62e^+^/AV^+^ (adjusted)	86.62 (69.05, 104.19)	59.61 (42.24, 76.97)	27.01 (2.00, 52.03) ^c^	0.035 ^d^

^a^ Mean (standard deviation); ^b^ Independent *t*-test applied; ^c^ Adjusted mean difference (95% confidence interval) with Bonferroni adjustment; ^d^ ANCOVA applied (adjusted for BMI and systolic blood pressure).

## Data Availability

Data are available from the corresponding author upon reasonable request.

## References

[1] Stanek A., Fazeli B., Bartuś S., Sutkowska E. (2018). The Role of Endothelium in Physiological and Pathological States: New Data. Biomed Res. Int..

[2] Dubois-deruy E., Peugnet V., Turkieh A., Pinet F. (2020). Oxidative Stress in Cardiovascular Diseases. Antioxidants.

[3] Jakubiak G.K., Cie G. (2022). Nitrotyrosine, Nitrated Lipoproteins, and Cardiovascular Dysfunction in Patients with Type 2 Diabetes: What Do We Know and What Remains to Be Explained ?. Antioxidants.

[4] Senoner T., Dichtl W. (2019). Oxidative Stress in Cardiovascular Diseases: Still a Therapeutic Target?. Nutrients.

[5] Jakubiak G.K., Osadnik K., Lejawa M., Osadnik T., Goławski M., Lewandowski P., Pawlas N. (2022). “Obesity and Insulin Resistance” Is the Component of the Metabolic Syndrome Most Strongly Associated with Oxidative Stress. Antioxidants.

[6] Celermajer D.S., Sorensen K.E., Gooch V.M., Miller O.I., Sullivan I.D., Lloyd J.K., Deanfield J.E., Spiegelhalter D.J. (1992). Non-Invasive Detection of Endothelial Dysfunction in Children and Adults at Risk of Atherosclerosis. Lancet.

[7] Xu S., Ilyas I., Little P.J., Li H., Kamato D., Zheng X., Luo S., Li Z., Liu P., Han J. (2021). Endothelial Dysfunction in Atherosclerotic Cardiovascular Diseases and beyond: From Mechanism to Pharmacotherapies. Pharmacol. Rev..

[8] Lugo-Gavidia L.M., Burger D., Matthews V.B., Nolde J.M., Galindo Kiuchi M., Carnagarin R., Kannenkeril D., Chan J., Joyson A., Herat L.Y. (2021). Role of Microparticles in Cardiovascular Disease: Implications for Endothelial Dysfunction, Thrombosis, and Inflammation. Hypertension.

[9] Vítková V., Živný J., Janota J. (2018). Endothelial Cell-Derived Microvesicles: Potential Mediators and Biomarkers of Pathologic Processes. Biomark. Med..

[10] Storch A.S., de Mattos J.D., Alves R., dos Santos Galdino I., Rocha H.N.M. (2017). Methods of Endothelial Function Assessment: Description and Applications. Int. J. Cardiovasc. Sci..

[11] Silambanan S., Hermes R.S., Bhaskar E., Gayathri S. (2020). Endothelial Microparticle as an Early Marker of Endothelial Dysfunction in Patients with Essential Hypertension: A Pilot Study. Indian J. Clin. Biochem..

[12] Leite A.R., Borges-Canha M., Cardoso R., Neves J.S., Castro-Ferreira R., Leite-Moreira A. (2020). Novel Biomarkers for Evaluation of Endothelial Dysfunction. Angiology.

[13] Boulanger C.M., Loyer X., Rautou P.E., Amabile N. (2017). Extracellular Vesicles in Coronary Artery Disease. Nat. Rev. Cardiol..

[14] Chen Y., Li G., Liu M.L. (2018). Microvesicles as Emerging Biomarkers and Therapeutic Targets in Cardiometabolic Diseases. Genom. Proteom. Bioinforma.

[15] Latham S.L., Tiberti N., Gokoolparsadh N., Holdaway K., Couraud P.O., Grau G.E.R., Combes V. (2015). Immuno-Analysis of Microparticles: Probing at the Limits of Detection. Sci. Rep..

[16] Paudel K.R., Panth N., Kim D.-W. (2016). Circulating Endothelial Microparticles: A Key Hallmark of Atherosclerosis Progression. Scientifica.

[17] Stockelman K.A., Hijman J.G., Bammet T.D., Greiner J.J., Stauffer B.L., DeSouza C.A. (2021). Circulating Endothelial Cell-Derived Microvesicles Are Elevated with Hypertension and Associated with Endothelial Dysfunction. Can. J. Physiol. Pharmacol..

[18] Sansone R., Baaken M., Horn P., Schuler D., Westenfeld R., Amabile N., Kelm M., Heiss C. (2018). Endothelial Microparticles and Vascular Parameters in Subjects with and without Arterial Hypertension and Coronary Artery Disease. Data Br..

[19] de Hoog V.C., Timmers L., Schoneveld A.H., Wang J.W., Van de Weg S.M., Sze S.K., Van Keulen J.K., Hoes A.W., Den Ruijter H.M., de Kleijn D.P. (2013). Serum Extracellular Vesicle Protein Levels Are Associated with Acute Coronary Syndrome. Eur. Heart J. Acute Cardiovasc. Care.

[20] Simak J., Gelderman M.P., Yu H., Wright V., Baird A.E. (2006). Circulating Endothelial Microparticles in Acute Ischemic Stroke: A Link to Severity, Lesion Volume and Outcome. J. Thromb. Haemost..

[21] Deng F., Wang S., Zhang L. (2017). Endothelial Microparticles Act as Novel Diagnostic and Therapeutic Biomarkers of Circulatory Hypoxia-Related Diseases: A Literature Review. J. Cell. Mol. Med..

[22] Tramontano A.F., Lyubarova R., Tsiakos J., Palaia T., Deleon J.R., Ragolia L. (2010). Circulating Endothelial Microparticles in Diabetes Mellitus. Mediat. Inflamm..

[23] Esposito K., Ciotola M., Schisano B., Gualdiero R., Sardelli L., Misso L., Giannetti G., Giugliano D. (2006). Endothelial Microparticles Correlate with Endothelial Dysfunction in Obese Women. J. Clin. Endocrinol. Metab..

[24] Jansen F., Li Q., Pfeifer A., Werner N. (2017). Endothelial- and Immune Cell-Derived Extracellular Vesicles in the Regulation of Cardiovascular Health and Disease. JACC Basic Transl. Sci..

[25] Berezin A.E., Berezin A.A. (2020). Endothelial Cell-Derived Extracellular Vesicles in Atherosclerosis: The Emerging Value for Diagnosis, Risk Stratification and Prognostication. Vessel Plus.

[26] Stoner L., Young J.M., Fryer S. (2012). Assessments of Arterial Stiffness and Endothelial Function Using Pulse Wave Analysis. Int. J. Vasc. Med..

[27] Amabile N., Guérin A.P., Leroyer A., Mallat Z., Nguyen C., Boddaert J., London G.M., Tedgui A., Boulanger C.M. (2005). Circulating Endothelial Microparticles Are Associated with Vascular Dysfunction in Patients with End-Stage Renal Failure. J. Am. Soc. Nephrol..

[28] Sansone R., Baaken M., Horn P., Schuler D., Westenfeld R., Amabile N., Kelm M., Heiss C. (2018). Release of Endothelial Microparticles in Patients with Arterial Hypertension, Hypertensive Emergencies and Catheter-Related Injury. Atherosclerosis.

[29] Chowienczyk P.J., Watts G.F., Cockcroft J.R., Ritter J.M. (1992). Impaired Endothelium-Dependent Vasodilation of Forearm Resistance Vessels in Hypercholesterolaemia. Lancet.

[30] Hermida N., Balligand J.L. (2014). Low-Density Lipoprotein-Cholesterol-Induced Endothelial Dysfunction and Oxidative Stress: The Role of Statins. Antioxid. Redox Signal..

[31] Schiffrin E.L. (2012). Vascular Remodeling in Hypertension: Mechanisms and Treatment. Hypertension.

[32] Jy W., Horstman L.L., Jimenez J.J., Ahn Y.S., Biró É., Nieuwland R., Sturk A., Dignat-George F., Sabatier F., Camoin-Jau L. (2004). Measuring Circulating Cell-Derived Microparticles. J. Thromb. Haemost..

[33] Navasiolava N.M., Dignat-George F., Sabatier F., Larina I.M., Demiot C., Fortrat J.O., Gauquelin-Koch G., Kozlovskaya I.B., Custaud M.A. (2010). Enforced Physical Inactivity Increases Endothelial Microparticle Levels in Healthy Volunteers. Am. J. Physiol. Heart Circ. Physiol..

[34] Lacroix R., Judicone C., Mooberry M., Boucekine M., Key N.S., Dignat-George F., Ambrozic A., Bailly N., Buffat C., Buzas E. (2013). Standardization of Pre-Analytical Variables in Plasma Microparticle Determination: Results of the International Society on Thrombosis and Haemostasis SSC Collaborative Workshop. J. Thromb. Haemost..

[35] Dey-Hazra E., Hertel B., Kirsch T., Woywodt A., Lovric S., Haller H., Haubitz M., Erdbruegger U. (2010). Detection of Circulating Microparticles by Flow Cytometry: Influence of Centrifugation, Filtration of Buffer, and Freezing. Vasc. Health Risk Manag..

[36] van Ierssel S.H., Van Craenenbroeck E.M., Conraads V.M., Van Tendeloo V.F., Vrints C.J., Jorens P.G., Hoymans V.Y. (2010). Flow Cytometric Detection of Endothelial Microparticles (EMP): Effects of Centrifugation and Storage Alter with the Phenotype Studied. Thromb. Res..

[37] Bratseth V., Margeirsdottir H.D., Chiva-Blanch G., Heier M., Solheim S., Arnesen H., Dahl-Jørgensen K., Seljeflot I. (2020). Annexin V+ Microvesicles in Children and Adolescents with Type 1 Diabetes: A Prospective Cohort Study. J. Diabetes Res..

[38] Bratseth V., Chiva-Blanch G., Byrkjeland R., Solheim S., Arnesen H., Seljeflot I. (2019). Elevated Levels of Circulating Microvesicles in Coronary Artery Disease Patients with Type 2 Diabetes and Albuminuria: Effects of Exercise Training. Diabetes Vasc. Dis. Res..

[39] Wilkinson I.B., Hall I.R., Maccallum H., Mackenzie I.S., Mceniery C.M., van der Arend B.J., Shu Y., Mackay L.S., Webb D.J., Cockcroft J.R. (2002). Pulse-Wave Analysis: Clinical Evaluation of a Noninvasive, Widely Applicable Method for Assessing Endothelial Function. Arterioscler. Thromb. Vasc. Biol..

[40] Ibrahim N.N.I.N., Rasool A.H.G. (2017). Assessment of Macrovascular Endothelial Function Using Pulse Wave Analysis and Its Association with Microvascular Reactivity in Healthy Subjects. Ski. Res. Technol..

[41] Greig L.D., Leslie S.J., Gibb F.W., Tan S., Newby D.E., Webb D.J. (2005). Comparative Effects of Glyceryl Trinitrate and Amyl Nitrite on Pulse Wave Reflection and Augmentation Index. Br. J. Clin. Pharmacol..

[42] Ibrahim N.N.I.N., Rasool A.H.G., Wong A.R., Rahman A.R.A. (2007). Methods Optimization to Assess Endothelial Function in Females- Duration of Glyceryl Trinitrate Effect. Methods Find. Exp. Clin. Pharmacol..

[43] Steinberg H.O., Bayazeed B., Hook G., Johnson A., Cronin J., Baron A.D. (1997). Endothelial Dysfunction Is Associated with Cholesterol Levels in the High Normal Range in Humans. Circulation.

[44] Torella D., Ellison G.M., Torella M., Vicinanza C., Aquila I., Iaconetti C., Scalise M., Marino F., Henning B.J., Lewis F.C. (2014). Carbonic Anhydrase Activation Is Associated with Worsened Pathological Remodeling in Human Ischemic Diabetic Cardiomyopathy. J. Am. Heart Assoc..

[45] Esposito K., Ciotola M., Sasso F.C., Cozzolino D., Saccomanno F., Assaloni R., Ceriello A., Giugliano D. (2007). Effect of a Single High-Fat Meal on Endothelial Function in Patients with the Metabolic Syndrome: Role of Tumor Necrosis Factor-α. Nutr. Metab. Cardiovasc. Dis..

[46] Suades R., Padró T., Alonso R., Mata P., Badimon L. (2013). Lipid-Lowering Therapy with Statins Reduces Microparticle Shedding from Endothelium, Platelets and Inflammatory Cells. Thromb. Haemost..

[47] Lacroix R., Robert S., Poncelet P., Kasthuri R.S., Key N.S., Dignat-George F. (2010). Standardization of Platelet-Derived Microparticle Enumeration by Flow Cytometry with Calibrated Beads: Results of the International Society on Thrombosis and Haemostasis SSC Collaborative Workshop. J. Thromb. Haemost..

[48] Dignat-George F., Boulanger C.M. (2011). The Many Faces of Endothelial Microparticles. Arterioscler. Thromb. Vasc. Biol..

[49] Dignat-George F. (2018). Extracellular Vesicles: Overview and Clinical Implications. Blood.

[50] Ståhl A.L., Johansson K., Mossberg M., Kahn R., Karpman D. (2019). Exosomes and Microvesicles in Normal Physiology, Pathophysiology, and Renal Diseases. Pediatr. Nephrol..

[51] Sharma R., Huang X., Brekken R.A., Schroit A.J. (2017). Detection of Phosphatidylserine-Positive Exosomes for the Diagnosis of Early-Stage Malignancies. Br. J. Cancer.

[52] Nguyen D.B., Thuy Ly T.B., Wesseling M.C., Hittinger M., Torge A., Devitt A., Perrie Y., Bernhardt I. (2016). Characterization of Microvesicles Released from Human Red Blood Cells. Cell. Physiol. Biochem..

[53] Brodsky S.V., Zhang F., Nasjletti A., Goligorsky M.S. (2004). Endothelium-Derived Microparticles Impair Endothelial Function In Vitro. Am. J. Physiol. Heart Circ. Physiol..

[54] Pirro M., Schillaci G., Paltriccia R., Bagaglia F., Menecali C., Mannarino M.R., Capanni M., Velardi A., Mannarino E. (2006). Increased Ratio of CD31+/CD42− Microparticles to Endothelial Progenitors as a Novel Marker of Atherosclerosis in Hypercholesterolemia. Arterioscler. Thromb. Vasc. Biol..

[55] Northrop E.F., Milbauer L.C., Rudser K.D., Fox C.K., Solovey A.N., Kaizer A.M., Hebbel R.P., Kelly A.S., Ryder J.R. (2019). Reproducibility of Endothelial Microparticles in Children and Adolescents. Biomark. Med..

[56] Pfrieger F.W., Vitale N. (2018). Cholesterol and the Journey of Extracellular Vesicles. J. Lipid Res..

[57] Fu Y., Luo N., Lopes-Virella M.F. (2000). Oxidized LDL Induces the Expression of ALBP/AP2 MRNA and Protein in Human THP-1 Macrophages. J. Lipid Res..

[58] Nomura S., Shouzu A., Omoto S., Nishikawa M., Iwasaka T. (2004). Effects of Losartan and Simvastatin on Monocyte-Derived Microparticles in Hypertensive Patients with and Without Type 2 Diabetes Mellitus. Clin. Appl. Thromb..

[59] Chiva-Blanch G., Badimon L. (2019). Cross-Talk between Lipoproteins and Inflammation: The Role of Microvesicles. J. Clin. Med..

[60] Guerrero F., Carmona A., Obrero T., Jiménez M.J., Soriano S., Moreno J.A., Martín-Malo A., Aljama P. (2020). Role of Endothelial Microvesicles Released by P-Cresol on Endothelial Dysfunction. Sci. Rep..

[61] Marei I., Chidiac O., Thomas B., Pasquier J., Dargham S., Robay A., Vakayil M., Jameesh M., Triggle C., Rafii A. (2022). Angiogenic Content of Microparticles in Patients with Diabetes and Coronary Artery Disease Predicts Networks of Endothelial Dysfunction. Cardiovasc. Diabetol..

[62] Venable A.S., Williams R.R., Haviland D.L., McFarlin B.K. (2014). An Analysis of Endothelial Microparticles as a Function of Cell Surface Antibodies and Centrifugation Techniques. J. Immunol. Methods.

